# Opto-Electric Cellular Biosensor Using Optically Transparent Indium Tin Oxide (ITO) Electrodes

**DOI:** 10.3390/s8053257

**Published:** 2008-05-19

**Authors:** Chang K. Choi, Chuck H. Margraves, Seung I. Jun, Anthony E. English, Philip D. Rack, Kenneth D. Kihm

**Affiliations:** 1 The University of Tennessee, Dept. of Mechanical, Aerospace, and Biomedical Engineering, Knoxville, TN 37996 USA; Chang K. Choi, Presently at Oak Ridge National Laboratory, Bioscience Division, Oak Ridge, TN 37831 USA; 2 The University of Tennessee, Dept. of Material Science and Engineering, Knoxville, TN 37996 USA; Seung I. Jun, Presently at dpiX, LLC, Process Engineering Group, Colorado Springs, CO 80916 USA

**Keywords:** Biosensors, Indium Tin Oxide (ITO), *In Vitro* Cytotoxicity, Optical Imaging, Micro-Impedance Detection, and Endothelial Cells

## Abstract

Indium tin oxide (ITO) biosensors are used to perform simultaneous optical and electrical measurements in order to examine the dynamic cellular attachment, spreading, and proliferation of endothelial cells (ECs) as well as cytotoxic effects when exposed to cytochalasin D. A detailed description of the fabrication of these sensors is provided and their superior optical characteristics are qualitatively shown using four different microscopic images. Differential interference contrast microscopy (DICM) images were acquired simultaneously with micro-impedance measurements as a function of frequency and time. A digital image processing algorithm quantified the cell-covered electrode area as a function of time. In addition, cytotoxicity effects, produced by the toxic agent cytochalasin D, were examined using micro-impedance measurements, confocal microscopy images of stained actin-filaments, and interference reflection contrast microscopy (IRCM) capable of examining the bottom morphology of a cell. The results of this study show (1) the dynamic optical and electrical cellular characteristics using optically thin ITO biosensors; (2) qualitative agreement between cell-covered electrode area and electrical impedance during cellular attachment; (3) in vitro cytotoxicity detection of ECs due to 3 μM cytochalasin D. The present opto-electric biosensor system is unique in that a simultaneous and integrated cellular analysis is possible for a variety of living cells.

## 1. Introduction

Indium oxide (In_2_O_3_) doped with tin oxide (SnO_2_), or Indium tin oxide (ITO), is an optically thin and electrically conductive material that is commonly used to make thin film layers on transparent conductive coatings for touch panel contacts, electrodes for liquid crystal and plasma displays, gas sensors, and solar cells. A new area where the benefit of being electrically conductive and optically transparent is important is in the dynamic examination of live cells under various environments.

Cellular micro-impedance measurements have found extensive application in quantifying cellular adhesion and barrier function. A relatively recent method, referred to as Electrical Cell-Substrate Impedance Sensing (ECIS) pioneered by Giaver and Keese [1-4], has become increasingly important in the study of cellular physiology [5-7]. This biosensor is based on a gold two-electrode configuration that consists of a small working electrode and a larger counter electrode. Although the information obtained from micro-impedance measurements using this gold electrode configuration compliment many existing optical microscopy techniques, it is a complicated and sensitive function of the cellular state in terms of cell-cell adhesion, cell-matrix adhesion, and cellular membrane properties. In other words, the measured electrical impedance is a function of the cellular morphology, cell-matrix attachment, and the degree of cell-cell contacts. However, the optical properties of gold limit its ability to perform simultaneous electrical impedance and microscopy measurements. Combined optical and micro-impedance measurements, therefore, have the potential to elucidate a number of complex cellular processes that optical and electrical measurements are not capable of independently. Despite both the optical and electrical benefits of ITO electrodes, to date few studies have examined the performance of ITO electrodes [8-11].

This study describes the fabrication and optical and electrical characteristics of ITO electrodes. Also presented is a digital image analysis of evolutionary images of live porcine pulmonary artery endothelial cells (PPAECs) on ITO electrodes in correlation with the simultaneously measured micro-impedance profiles. Furthermore, the time dependent cellular impedance response to 3 μM Cytochalasin D (a toxic agent) at 5.62 kHz and confocal images of stained fixed endothelial cells (ECs) as well as interference reflection contrast microscopy (IRCM) images of live ECs further demonstrate the ability to combine electrical micro-impedance measurements with microscopy methods for a number of opportunities for the study of cellular physiology.

## 2. Methods and Materials

### 2.1. Indium Tin Oxide Silicon Nitride (ITO-Si_3_N_4_) Electrode Fabrication

**Figure 1.**
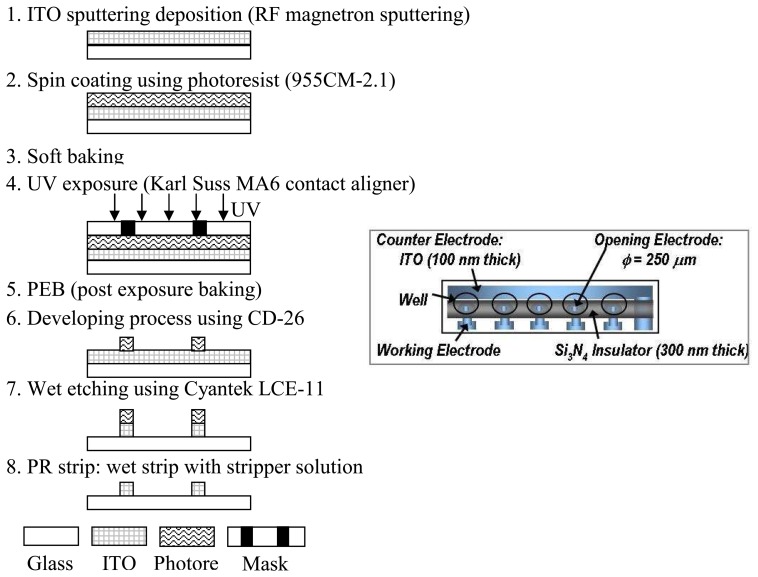
Fabrication of an ITO thin film microelectrode on a slide glass. A 90 % In_2_O_3_/10 %SnO_2_ 100 nm layer was sputter coated onto a slide glass. Using standard photolithography methods, an array of five ITO electrodes and a single counter electrode was developed. The schematic in the box shows the top view of the ITO- Si_3_N_4_ electrode.

**Figure 2.**
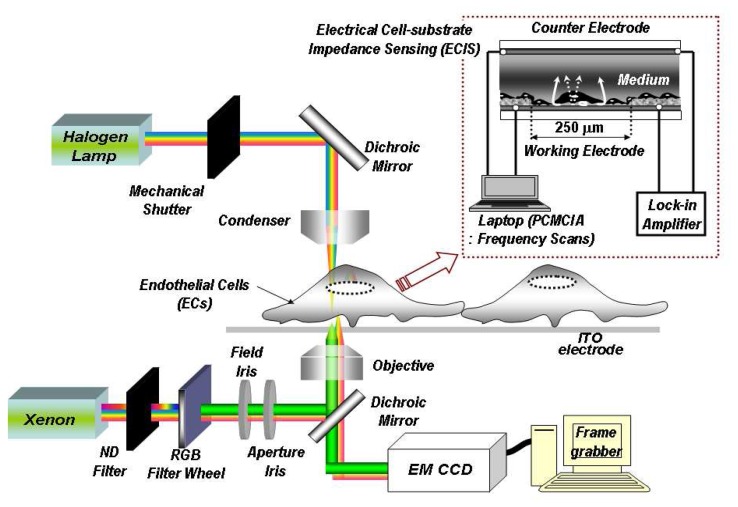
Experimental schematic of a dynamic integrated opto-electric system having electrical impedance measurement, transmitted DICM imaging, and multi-spectrum IRCM imaging.

**Figure 3.**
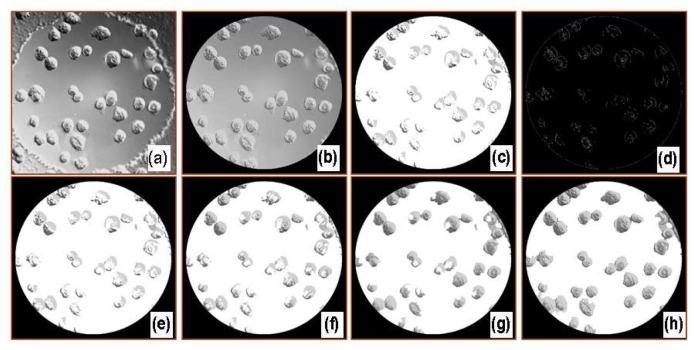
Digitally processed images produced by each filter. To automate the cell covered area estimation, a sequence of image processing steps is carried out. (a) Original image. (b) Deconvolved image with examined area outside electrode set to zero. (c) After applying threshold filtering with high and low threshold limits. (d) Binary image created using a Canny filter. (e) Image after combining (c) and (d). (f) After applying diagonal filter along the shear axis. (g) After the filling filter. (h) After removal filter.

**Figure 4.**
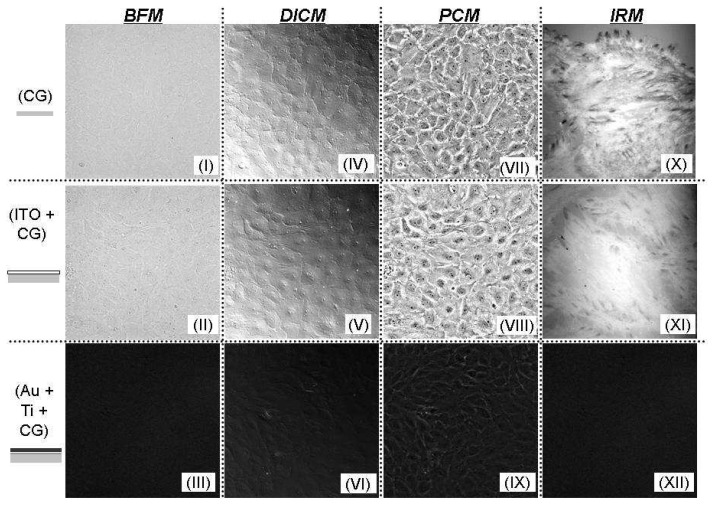
Various microscopic images of PPAECs cultivated on a glass substrate, a glass surface coated with a 100 nm thick ITO film, and a glass surface coated with a Ti (2.5 nm)/Au (47.5 nm) layer. All the microscopic images through the ITO film are comparable to those of a bare glass surface.

**Figure 5.**
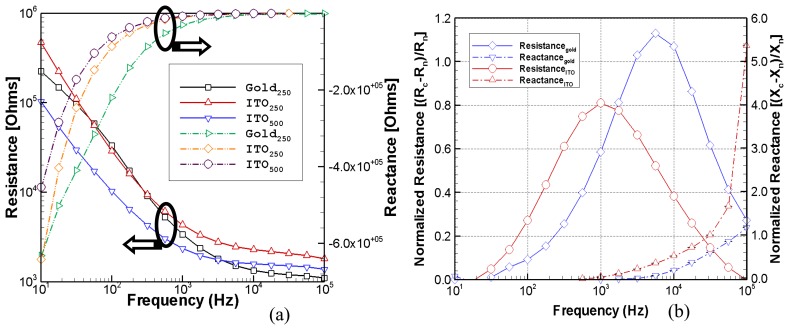
(a) Real and imaginary frequency dependent impedance response of naked 250 μm diameter gold electrodes and 250 and 500 μm diameter ITO electrodes. (b) Frequency dependent normalized impedance of 250 μm gold and ITO electrodes: The terms *R* and *X* represent the resistance and reactance, respectively and the subscripts *c* and *n* indicate cell covered and naked scans, respectively.

**Figure 6.**
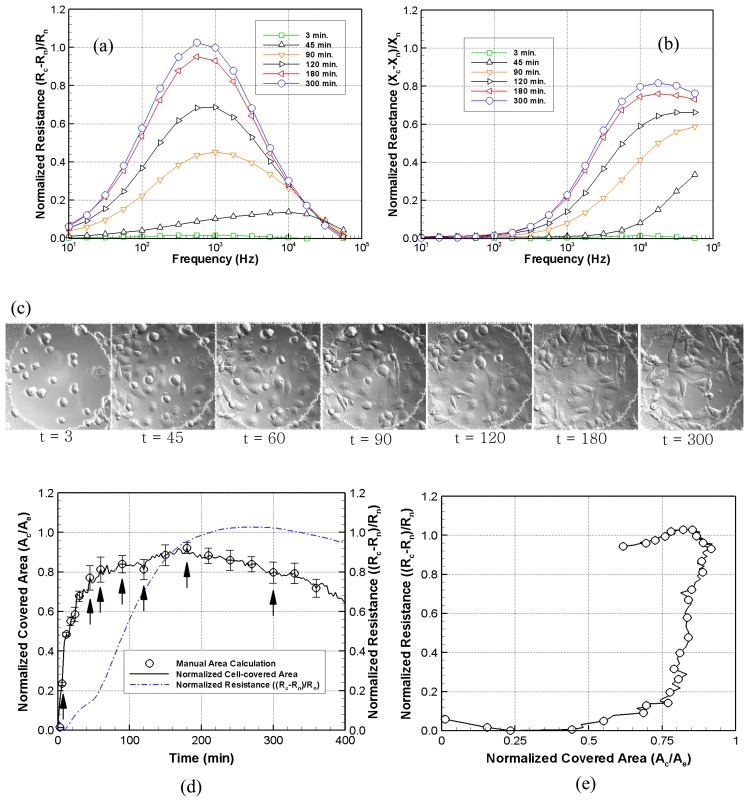
Concurrent normalized impedance, cell covered area and DICM images of attaching PPEACs at *t* = 3 min, 45 min, 60 min, 90 min, 120 min, 180 min, and 300 min. (a) The normalized resistance as a function of frequency shows a monotonic increase with increasing time up to 300 minutes. (b) The corresponding normalized reactance also shows an increase with increasing time during the cellular attachment phase. (c) The corresponding DICM images illustrate the changes in the cell covered area and flattening cell morphology with increasing time. (d) The normalized cell-covered area and the normalized resistance both initially increase with time and then gradually decrease. (e) The normalized resistance as a function of the normalized cell-covered area does not show a linearly proportional relationship.

**Figure 7.**
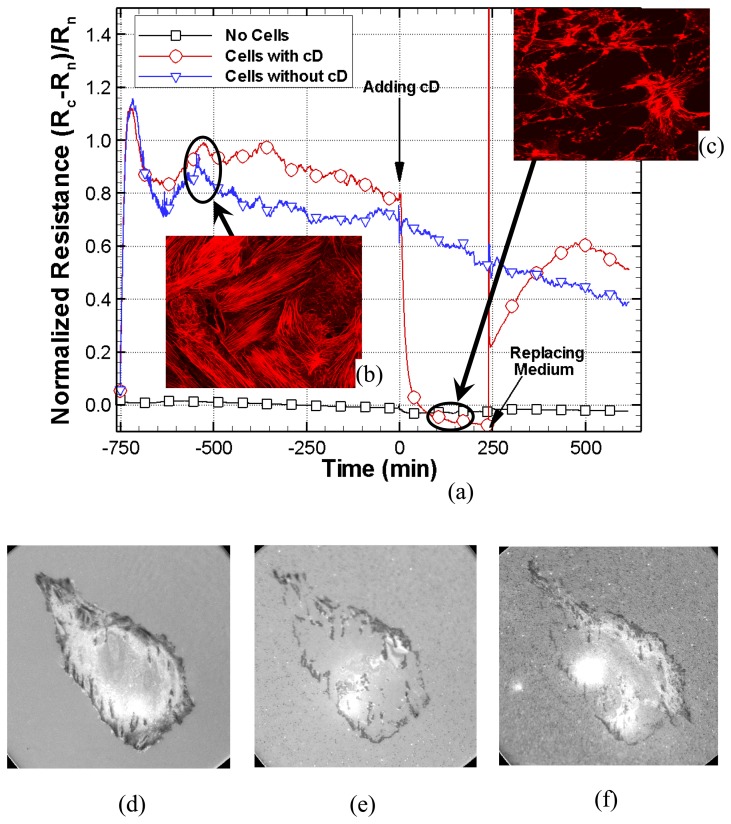
Three methods to examine the cytotoxic effects of cytochalasin D on PPAECs. (a) Normalized resistance response. Data were obtained every 1.2 second, however in order to distinguish lines, only 20 data points were marked. Stained confocal images (b, c) of actin-filaments. Corresponding IRCM images of bottom morphology changes, 10 minutes before adding cytochalasin D (d), 240 minutes after adding cytochalasin D (e), and another 240 minutes after replacing the treated medium with new complete medium (f).

Figure 1 summarizes the ITO electrode fabrication process. The ITO biosensor array consisted of six 100 nm ITO film electrodes deposited on a 76 mm × 26 mm glass slide using an AJA ATC2000 RF magnetron sputtering system (Fig. 1-1). Five of the electrodes were used as working electrodes while the sixth was used as a common counter electrode. The sputtering target consisted of 90wt. % In_2_O_3_ and 10wt. % SnO_2_. The base pressure prior to the sputtering deposition was below 5.3 μPa. During sputter deposition, a flow rate of 25 sccm of Ar-H_2_ was introduced into the chamber to produce a 3 mTorr pressure. The ITO sputtering power was 200 W and the film was deposited for 15 minutes at a temperature of 400 °C. Subsequent to the ITO thin film sputtering, photoresist (955CM-1.1) was spin-coated for 50 seconds at 3000 rpm resulting in a nominal 1.5 μm thickness (Fig. 1-2). After spin coating, the photoresist was soft baked at 90 °C for 45 seconds (Fig. 1-3), exposed to 365 nm wavelength light for 1.5 seconds using a Karl Suss MA6 contact lithography system (Fig. 1-4), post exposure baked at 120 °C for 45 seconds (Fig. 1-5), and developed in CD-26 developer for 70 seconds (Fig. 1-6). Finally, the ITO was wet etched with Cyantek LCE-11 etchant at 40 °C (Fig. 1-7). Stripper solution then removed the photoresist at 70 °C (Fig. 1-8).

On the top of the patterned ITO electrode, RF magnetron sputtering in argon-hydrogen (95% Ar - 5% H_2_) and nitrogen gases deposited an insulating 300nm silicon nitride film. The sputter deposition condition of the Silicon Nitride film was 100 W RF power, 5 mTorr pressure, 25 sccm Ar-H_2_, and a 25 sccm N_2_ gas flow at a temperature of 300 °C. A similar photolithography process as described for the ITO film pattern produced the silicon nitride layer. The silicon nitride insulating wells on the array were patterned by reactive ion etching (RIE) with SF6/O2 chemistry.

Because the working electrode constrictive impedance dominates the impedance of the entire system, it is possible to detect the cellular impedance. The Silicon nitride (Si_3_N_4_) layer is resistant to ethanol sterilization and therefore all electrodes were sterilized with a 70 % ethanol 30 % de-ionized (DI) water solution and then rinsed with sterilized DI water.

### 2.2. Integrated Dynamic Live Cell Imaging System

Figure 2 shows an experimental schematic of an integrated dynamic opto-electric apparatus. An SR830 lock-in amplifier circuit generated a current through the ITO electrode and measured the resulting electrode voltage. A computer equipped with a PCMCIA card and a LabView based data acquisition program controlled the amplifier and performed frequency scans. Cells were kept viable using an incubator (WeatherStation, Olympus) that kept the temperature (37°C), humidity, and CO_2_ (5%) levels constant. These controlled conditions were crucial to keep cells alive during long time lapse measurements. The imaging system consisted of a 20x phase contrast microscopic (PCM) objective, a 20x plan semi-apochromat objective, a 100x oil-immersion plan semi-apochromat objective, corresponding PCM, DICM, and IRCM optical components on an Olympus Model IX-71 inverted microscope, and a Hamamatsu 14-bit electron multiplier (EM) cooled and intensified-CCD digital camera. Additionally, a mechanical shutter was synchronized with the CCD camera to minimize any effects the halogen lamp may have had on cell growth [9, 12, 13].

### 2.3. Digital Imaging Processing

Figure 3 shows digitally processed images through each filter. A deconvolved image (b) was first obtained using a Gaussian point spread function. The following image, (c), was obtained after removing a portion of the background from the cell-covered areas using high and low threshold limits, while a complete binary image, (d), was separately obtained using a Canny or Sobel edge detection filter. The local gradients were compared to high and low threshold values, either provided by the user or internally calculated, to roughly detect the cell boundary. A pixel by pixel comparison of images (c) and (d) was then made to more accurately define the cell membrane boundaries. Generally, a portion of each cell was removed using the threshold filter as some of these pixels had similar intensities compared to those of the surrounding medium. A general characteristic of DICM is that the intensities in a cell varied from darker to brighter or brighter to darker along the shear axis. The diagonal filter compensated for this eliminated area and specified single cells. Another filter, referred to as a stitch filter, was employed to completely fill holes in cells to produce image (g). Finally, a removal filter deleted the defects that appeared as cells but were possibly small air bubbles or optical artifacts. The last image, (h), gives the area covered by cells using the image processing algorithm. An additional step may be used to obtain overlay images which were used to visually check cell-occupied areas overestimated or discarded from digital image processing

### 2.4. Cellular Micro-Impedance Measurement

A data acquisition and analysis system was implemented using LabVIEW software. A reference voltage source provided an ac 1v_rms_ reference signal via a series 1 MΩ resistor to the electrode array. A National Instruments SCXI-1331 switch made successive connections between the different working electrodes and the counter electrode of each array. The source voltage generator resistance was 50 Ω. Because the series resistance was significantly larger the electrode impedance over the range of frequencies used in this study, an approximately constant current of 1 μA was provided to the electrode. An SR830 lock-in amplifier measured the electrode voltage. The input impedance of the lock-in amplifier was equivalent to a parallel resistor and capacitor combination of 10 MΩ and 10 pF, respectively. Direct measurements of the cable parasitic capacitances were made using an LCR meter and incorporated into a circuit model to estimate the impedance based on the lock-in voltage measurement.

A total of 17 evenly spaced logarithmic frequencies between 10 Hz and 100 kHz were chosen. Preliminary naked scans were performed to optimize the sensitivity at each frequency. The preliminary naked scan checked for any debris on the ITO-Si3N4 electrodes as well as electrode defects. A 1.2 second naked scan sampled at a rate of 32 Hz was then performed at each frequency for the naked electrodes. The electrodes were then inoculated with 400 μL porcine pulmonary artery endothelial cells (PPAECs) and medium. During the cellular attach scans, data was acquired at a rate of 32 Hz using a 30 ms filter time constant and 12 dB/decade roll off.

### 2.5. Cell Culture

Endothelial cells were isolated from porcine pulmonary arteries obtained from a local abattoir. The endothelial cells were cultivated in an incubator at 37 °C and 5 % CO_2_. The cell culture media consisted of M199 (GibcoBRL) and 10 % fetal bovine serum (Hyclone) supplemented with BME vitamins (Sigma), L-glutamine (GibcoBRL), penicillin and streptomycin (GibcoBRL), and BME amino acids (Sigma). The culture was maintained for approximately one week at which point the cells reached confluence. They were then regularly passaged once a week. For this study, passages between four and ten were used for measurements. Trypsin-EDTA (1X, GibcoBRL) was used to detach cells for passaging and electrode inoculation. Endothelial cells suspended in M199 were inoculated directly onto a series of sterilized ITO-Si_3_N_4_ microelectrodes that were not previously coated with any adhesion molecules such as fibronectin.

## 3. Results

### 3.1. Optical Properties of Gold and ITO Electrodes

Figure 4 shows various microscopic images of PPAECs cultivated on a glass substrate, a glass surface coated with a 100 nm thick ITO film, and a glass surface coated with a Ti (2.5 nm)/Au (47.5 nm) layer. All the microscopic images through the ITO film clearly show that the high transmittance of an ITO electrode makes them comparable to images seen through bare glass. Images seen through the Au/Ti layer under an identical illumination condition, however, are imperceptible. The size of the images from (I) to (IX) is 400 μm × 400 μm, while that of IRM images is 80 μm × 80 μm. These qualitative characteristics show good agreement with quantitative measurements and analysis previously published [9]. Thus all the microscopic techniques, for example bright field microscopy (BFM), differential interference contrast microscopy (DICM), phase contrast microscopy (PCM), interference reflection microscopy (IRM), confocal microscopy, etc. can be employed for the simultaneous optical and electrical measurement of cellular behaviors on ITO electrodes.

### 3.2. Electrical Characteristics of ITO Electrodes

Figure 5 shows the electrical characteristics of ITO electrodes. Figure 5a compares the real and imaginary frequency dependent impedances of naked 250 μm diameter gold electrode and 250 μm and 500 μm diameter ITO electrodes. The 250 μm ITO electrode has the higher constrictive resistance and reactance, compared to the 500 μm. However, the 250 μm ITO silicon nitride electrode has higher resistance than the same size gold electrode especially at higher frequencies. Figure 5b shows the frequency dependent normalized resistance and reactance of a 250 μm diameter gold electrode and a 250 μm diameter ITO electrode during the cellular attachment of PPAECs. The normalized resistance, (*R_c_*-*R_n_*)/*R_n_*, and the normalized reactance, (*X_c_-X_n_*)*/X_n_*, were calculated from the cell covered and naked electrode resistances, *R_c_* and *R_n_*, and reactances, *X_c_* and *X_n_*, respectively. The measured resistance increases when the electrode is covered with cells, for both the gold and the ITO electrodes demonstrating the feasibility of using the ITO electrode as a bio-impedance sensor. The 250 μm ITO electrode shows similar but somewhat reduced sensitivity to endothelial cell attachment compared to the 250 μm gold electrode. The measurement sensitivity of the cell-covered electrode resistance is greatest in the range of 563 Hz to 5.62 kHz with a peak around 5.62 kHz for the gold electrode and 1.0 kHz for the ITO electrode.

### 3.3. Combined Electro-Optic Analysis

Figures 6a and 6b show normalized resistance and reactance attach scans as a function of frequency for times *t* = 3 min, 45 min, 60 min, 90 min, 120 min, 180 min, and 300 min. The normalized values for the resistance and reactance are determined using the relations: (*R_c_*-*R_n_*)*/R_n,_* and (*X_c_*-*X_n_*)*/X_n_*, respectively. Figure 6c presents the raw DIC images and Fig. 6d gives the normalized cell-covered area and the corresponding normalized resistance versus time lapse after image processing had been completed. Finally, Fig. 6e gives the corresponding normalized resistance as a function of normalized cell-covered area. The normalized resistance scans show a characteristic increase in the peak amplitude as the cells attach to the ITO electrode surface. The measurement sensitivity of the cell-covered electrode resistance in this case is highest around 562 Hz. The normalized changes in the reactance also exhibit an increase during the attachment process.

The solid line shown in Fig. 6d is the result of 140 images taken at a rate of 1 frame per 3 minutes for 7 hours. The symbols show selected data points whose area was both digitally and manually measured, in order to determine the effectiveness of the DIP software. Half of the error bars represent the area differences between the digital imaging processing and the manual tracking. It was determined that the DIP software was on average within 5% of the manual measurements. The arrows indicate corresponding points for selected resistances, reactances, and raw images. The normalized cell-covered area versus time shows that until the endothelial cells reach 95% confluence, the normalized resistance and reactance increase as the cell-covered area increases. However, it was determined that the resistance continued to increase after the cell-covered area decreased following confluence. The normalized cell-covered area at *t* = 300 min decreased by approximately 11 % compared with that at *t* = 180 min while the normalized resistance and reactance slightly increased due to other factors, such as cell-cell adhesion and cell-substrate interactions. These electrical changes in resistance and reactance can perhaps best be explained by optically examining the cells.

Figure 6e shows that the normalized resistance did not completely reflect the amount of the electrode covered with cells. Although the time dependent patterns shown in Fig. 6d are similar, the normalized cell-covered area remains constant while the normalized resistance continuously increased as a possible result of increasing cell-substrate and cell-cell adhesion. The cells appeared much flatter and more tightly packed in these cases. Changes in normalized resistance and reactance are a complicated function of the normalized cell-covered area, cell-cell adhesion, and cell-substrate adhesion. All of these factors can be considered both optically and electrically with a transparent conductive ITO electrode. Increasing cell-cell and cell-matrix adhesion states, however, are consistent with the more tightly packed and flatter cell morphologies. This confirms cellular attachment on ITO silicon nitride bioelectrodes and demonstrates the potential of ITO electrodes as a promising tool for both electrical measurement and optical visualization.

### 3.4. Endothelial Cell Response to Chytochalasin D on ITO Electrodes

Figure 7 presents three methods to examine the cytotoxic effects of cytochalasin D on endothelial cells. Fig. 7a shows the normalized resistance response of PPAECs on an ITO electrode when inoculated with 3 μM of Cytochalasin D. The resistance (black line) of naked scans, where no cells are present, remains constant while attached scans (blue line) under normal conditions show a constant decrease over time. However the addition of 3 μM of cytochalasin D shows an immediate decrease in the measured resistance. Note that there is no fluctuation in the naked scans and significantly less fluctuation after adding cytochalasin D, while attach scans under the normal cellular environmental conditions show relatively large fluctuations. There is also a distinctive peak due to the cellular attachment followed by decease in the normalized resistance in all the wells over the first several hours. Adding cytochalasin D produced a systematic decrease in the resistance and removal of the drug, achieved by replacing the medium, produced an abrupt increase.

After replacement, a second peak was observed in the impedance due to the reattachment of cells, followed by a steady decrease. Figures 7b and 7c show stained confocal images of actin-filaments before and after inoculation with cytochalasin D. These pictures provide clarity on how the actin filaments were disrupted under 3 μM of cytochalasin D which in turn explains the decrease in resistance. Figures 7d-7f show IRM images 10 minutes before adding cytochalasin D, 240 minutes after adding cytochalasin D, and another 240 minutes after replacing the treated medium with new complete medium. Note that these optical images are not simultaneously taken with micro-impedance measurement because they needed higher magnification objective lens which must not use slide-glasses but cover-slips. This result is consistent with micro-impedance measurement and stained confocal images. However unlike the other methods, IRCM is label free, clearly shows bottom morphology changes caused by the toxic agent, and has both time and spatial resolution for single cells.

## 4. Discussion

Micro-impedance measurement using gold electrodes have found widespread application in cellular biology where the continuous electrical tracking of cellular attachment, growth, proliferation, and micromotion is desired [14-16]. In particular, there has been extensive interest in the application of this electrical sensor to probing cellular responses to toxic and pharmacological agents [5, 17, 18]. Indium Tin Oxide has the potential to combine the attributes of gold microelectrodes with many forms of cellular microscopy. Cellular barrier function as measured by microelectrode impedance spectroscopy is a complicated and sensitive function of cell-cell, cell-matrix, and membrane impedance components. These adhesive and membrane properties are in turn regulated by the cellular cytoskeleton. As a result, the simultaneous visualization and measurement of barrier function and cellular cytoskeletal organization using ITO silicon nitride biosensors has a number of potential applications in cellular biology.

The optimal development of a transparent micro-impedance biosensor requires a number of important considerations. The naked electrode impedance will depend on the ITO thickness and the size of the microelectrode. In this study, the 250 μm and 500 μm electrodes enable endothelial cell barrier function to be measured as a function of time and frequency. The ITO bioelectrode also provides low roughness, ease of patterning, uniform transmission homogeneity, and resistance homogeneity.

Although electrical signals are extremely sensitive to the different stages of cellular attachment, they are a complicated function of the degree of cell-cell and cell-matrix interaction as well as the fraction of the covered electrode surface area. Several degenerate states, giving similar electrical impedances, can potentially arise under very different degrees of electrode coverage and states of cellular attachment. In some cases, for example, it is possible to have a small fraction of the electrode covered by tightly adhering cells that gives rise to a similar impedance measurement produced by a confluent covering of loosely attached cells. Simultaneous optical images can provide a direct measure of the fraction of the electrode covered by cells and, therefore, remove this ambiguity

Excellent resolution and improved cell boundary visibility can be obtained using DICM, particularly in the adjoining cellular regions that have larger optical gradients. In general, the use of a high numerical aperture objective lens provides optical slicing in a thick specimen. The shear axis, which is a chief characteristic in DICM and along which the maximum contrast exists, can be exploited in the application of image processing algorithms, particularly those that implement a diagonal filter. Annoying halos, encountered in phase contrast microscopy (PCM), are absent in DICM images. Plastic materials, such as tissue culture dishes, commercial gold electrode, and other birefringent specimens, however, are not suitable for DICM measurements because of their effect upon polarized light. Thus, in order to properly use the DICM technique, all the materials along the light path must consist of glass. The transparent conductive ITO-Si_3_N_4_ electrodes used in this experiment are sufficiently compatible with DICM imaging.

The use of an ITO electrode on a coverslip simultaneously allows optical IRM and electrical impedance measurements which can be used to examine the dynamic response of cells to different toxins or drugs. This may prove to be a valuable tool for many drug delivery systems and tissue engineering applications where cytotoxicity tests can determine the mechanism and structure of cells, such as division, proliferation, and migration, by analyzing the effect of the drug on actin filaments. The cellular attachment, such as the formation of cell-cell and cell-substrate contacts, gives rise to measured changes in the resistance and reactance.

The feasibility of using an optically thin ITO-Si_3_N_4_ electrode is demonstrated as an opto-electric biosensor to simultaneously produce dynamic images of the motion and growth of cells while concurrently measuring cellular impedance. Compared to the industry-standard gold electrode, the ITO biosensor provides superior optical transmittance that permits live cellular imaging while also being sufficiently sensitive to function properly as a micro-impedance sensor. Furthermore, the ITO electrode patterned by Si_3_N_4_ layer allows for repeated use as both materials (ITO and Si_3_N_4_) are undamaged by ethanol-cleaning or DI water-sterilization processes unlike standard gold electrodes which significantly deteriorate after repeated cleanings. Additionally, the ITO electrode is simpler to fabricate. Indium tin oxide can be directly layered on a coverglass substrate, while gold requires an additional metal-coated layer (Ti or Cr) to promote its adhesion to the glass surface. The present opto-electric biosensor system is unique in that a simultaneous and integrated cellular analysis is possible for a variety of living cells. Future work using this biosensor will include examining human colorectal cancer cells under various cytotoxic agents and drug treatments.

## 5. Conclusion

This study demonstrates the successful development of an optically thin and electrically conductive ITO electrode. The biosensor was used to perform simultaneous optical and electrical measurements in the examination of cellular proliferation and *in vitro* cytotoxicity. The fabrication process of indium tin oxide silicon nitride (ITO-Si_3_N_4_) electrode was explained in detail. Its optical and electrical characteristics of porcine pulmonary artery endothelial cells were qualitatively and quantitatively examined. Additionally electrical micro-impedance analysis of ECs under cytochalasin D showed good agreement with optical cellular images using confocal microscopy and interference reflection microscopy.
